# Case Report: Drug-induced Leukocytoclastic Vasculitis in a Patient with Classic Ehlers-Danlos Syndrome

**DOI:** 10.7759/cureus.5915

**Published:** 2019-10-15

**Authors:** Khalil Bourji, Mark Marchitto, Sumanth Kuppalli, Johnny Dang, Bupesh Dogra

**Affiliations:** 1 Rheumatology, Wayne State University, Detroit, USA; 2 Dermatology, Johns Hopkins Hospital, Baltimore, USA; 3 Anesthesiology, Beth Israel Deaconess Medical Center, Boston, USA; 4 Medicine, Trinity School of Medicine, Kingstown, VCT; 5 Medicine, Sinai Hospital of Baltimore, Baltimore, USA

**Keywords:** ehlers-danlos, vasculitis, sulfonamide, drug-induced, small-vessel vasculitis

## Abstract

Drug-induced skin reactions are common, but only a small portion (10%) are attributed to a vasculitic mechanism. Small-vessel vasculitis (SVV) with leukocytoclastic histopathology is usually described in drug-induced vasculitis; however, given the shared histopathologic features between drug-induced vasculitis and other SVV, it is crucial to rule out infectious or other autoimmune etiologies underlying the clinical presentation. We hereby sought to present a case of sulfonamide-induced leukocytoclastic vasculitis, limited to the skin, in a patient with Ehlers-Danlos syndrome in order to emphasize the need for a broad diagnostic and clinical exclusion workup.

## Introduction

Small-vessel vasculitis (SVV) is characterized by the inflammation of small vessels clinically manifesting as palpable purpura with a leukocytoclastic histopathologic pattern. SVV can be classified as primary/idiopathic or secondary, with common etiologies being infections, connective tissue disorders, adverse drug reactions, or malignancy. Drug-induced skin reactions are common, but only a small portion (~10%) are attributed to a vasculitic mechanism; hence, a definitive diagnosis of drug-induced vasculitis is often challenging and requires exclusion of other potential triggers [1]. We report a case of SVV thought to be induced by sulfonamide use in a patient with Ehlers-Danlos syndrome (EDS).

## Case presentation

**Figure 1:**
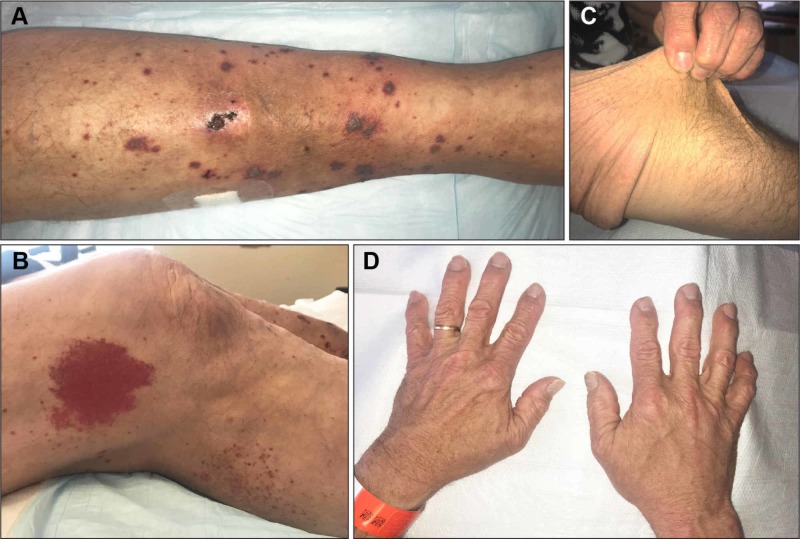
Clinical images A, Patient’s left leg with numerous non-blanching purpuric macules and patches with some ulceration noted. B, Large non-blanching purpuric patch on lateral aspect of right thigh representing a new vasculitic lesion. C, Skin hyperelasticity. D, Hands displaying subtle swan-neck deformities and thumb subluxation

**Figure 2:**
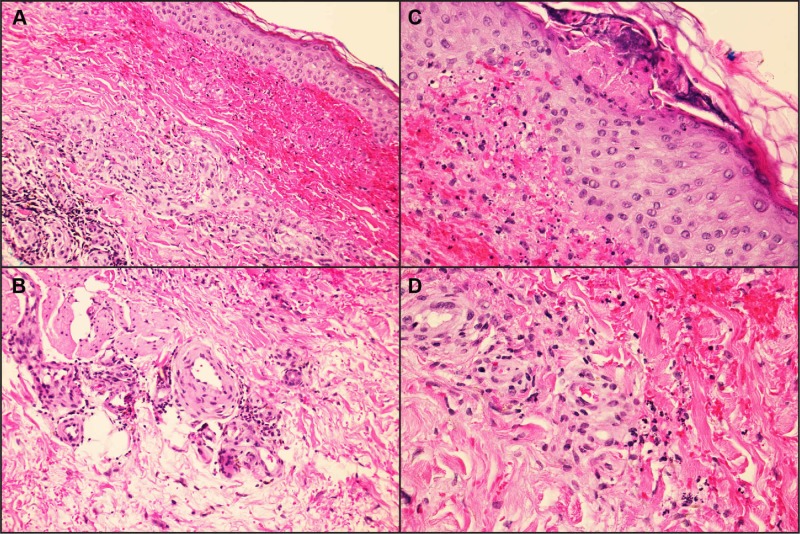
Histopathology of a lesion from the left lower extremity A and B, Superficial perivascular dermal infiltrate of neutrophils and eosinophils, with associated nuclear debris and extravasated red blood cells consistent with a diagnosis of leukocytoclastic vasculitis. C, Transepidermal disruption with extravasating keratin and underlying nuclear debris and hemorrhage. D, Multinucleated cells infiltrating small postcapillary venules and capillary loops in the papillary dermis (hematoxylin-eosin; magnification A ×200, B ×400, C ×400, D ×400).

A 63-year-old Caucasian man with a history of classic EDS type II “mitis,” frequent ecchymoses, and poor wound healing presented with a non-pruritic skin rash that appeared 24-48 hours after completing a seven-day course of oral sulfamethoxazole/trimethoprim for recently diagnosed cellulitis. The patient denied any fever, photosensitivity, urinary, or gastrointestinal symptoms. On clinical examination, multiple pinpoint to 2 mm red/purple non-blanching macules coalescing into purpuric plaques on lower and upper extremities were present (Figure 1A, 1B). In addition, hyperextensible skin was noted (Figure 1C). Laboratory findings, including differential blood count, comprehensive metabolic panel, urinalysis, and serum and urinary protein electrophoresis, were unremarkable. Serology for HIV, hepatitis B and C viruses, antinuclear antibody, double-strand DNA antibody, rheumatoid factor, antineutrophil cytoplasmic antibodies (C-ANCA and P-ANCA), antiribonucleic protein antibody, anti-SS-A, anti-SS-B, and cryoglobulin were also unremarkable. Skin punch biopsy (3mm) was performed on one of the left lower extremity macules, and the pathologic examination showed leukocytoclastic vasculitis (LCV) (Figure 2). During the hospital stay, the patient required supportive care only, and the offending drug was already discontinued prior to admission. The patient was discharged after an observation of 48 hours. On four weeks follow-up with his dermatologist, the patient skin rash was almost resolved with minor residuals.

## Discussion

LCV is considered the hallmark histopathologic pattern of SVV and is characterized by angiocentric segmental inflammation, endothelial cell swelling, erythrocyte extravasation, fibrinoid necrosis, and cellular infiltrates with mostly neutrophils of fragmented nuclei (karyorrhexis and leukocytoclasia) [2].

EDS is a heritable disorder of the connective tissue related to genetic defects that affect the biosynthesis and structure of collagen. EDS results in variable clinical manifestations but is classically characterized by skin hyperextensibility, joint hypermobility, and poor wound healing. Our patient had a molecularly confirmed "classical" EDS that is characterized by the reduction in the amount of type V collagen. Vascular complications of EDS tend to occur in arteries of large and medium caliber (e.g., proximal and distal branches of the aorta) where small-vessel involvement is unusual [3,4]. Typically, "vascular" EDS is caused by mutations in the gene that encodes the chains of type III collagen, which is the main protein of the walls of blood vessels [5]. Moreover, the presence of EDS with skin fragility and easy bruising may confuse clinicians in determining the etiology of a skin purpura, which in this case was independent from the EDS diagnosis. 

Drug-induced vasculitis should be considered in any patient with SVV, especially when confined to the skin. Many therapeutic agents, including sulfonamides, have been associated with vasculitis that can generally be categorized as ANCA-positive or ANCA-negative. ANCA-negative drug-induced vasculitis is usually confined to the skin alone and presents within days to weeks of exposure [1,6]. In this case, obtaining an extensive medication history and eliciting exposures to potential triggers was fundamental to diagnosis. The time frame correlation between the exposure to the offending drug and the onset of the skin purpura in addition to the exclusion of other potential infectious and autoimmune etiologies helped in establishing the diagnosis. 

## Conclusions

Finally, it is important to note the shared histopathologic features between drug-induced vasculitis and other SVV, making it necessary to exclude viral infections, such as HIV and viral hepatitis, and autoimmune conditions in these patients. Clearly, a comprehensive approach to the workup of LCV is critical because its management differs depending on the clinical context and underlying cause of the SVV.
